# Neurofibromatosis type 1-plexiform neurofibromas: Integrating treatment across pediatric and adult populations

**DOI:** 10.1093/neuonc/noag023

**Published:** 2026-02-05

**Authors:** Amy E Armstrong, Andrea M Gross, Laura J Klesse, Steven D Rhodes, Shivani Ahlawat, Verena Staedtke, Camilo A Molina, Angela C Hirbe

**Affiliations:** Division of Pediatric Hematology and Oncology, St. Louis Children’s Hospital, Washington University School of Medicine, St. Louis, Missouri, USA; Department of Pediatrics, University of Cincinnati College of Medicine, Cincinnati, Ohio, USA; University of Texas Southwestern/Children’s Health, Dallas, Texas, USA; Indiana University School of Medicine, Indianapolis, Indiana, USA; Johns Hopkins University School of Medicine, Baltimore, Maryland, USA; Johns Hopkins University School of Medicine, Baltimore, Maryland, USA; Division of Pediatric Hematology and Oncology, St. Louis Children’s Hospital, Washington University School of Medicine, St. Louis, Missouri, USA; Division of Oncology, Department of Medicine, Siteman Cancer Center, Barnes Jewish Hospital, Washington University School of Medicine, St. Louis, MO, USA

**Keywords:** MEK-inhibitor-targeted therapy, natural history, neurofibromatosis type 1, plexiform neurofibromas, therapeutics

## Abstract

Plexiform neurofibromas (PNs) are a hallmark of neurofibromatosis type 1 (NF1), affecting ∼50% of individuals with the condition. Originating from Schwann cells and other peripheral nerve sheath components, these tumors can cause significant morbidity, including functional impairment, diminished health-related quality of life, chronic pain, and malignant transformation. Managing NF1-PNs is challenging because of disease variability, differing growth rates, and age-related differences in clinical presentation and treatment tolerability. This review examines current therapeutic strategies, including surgery, medical therapies, and emerging treatments, emphasizing individualized care. Highlighted here is the need for age-specific treatment planning, particularly as disease progression, comorbidities, and side-effect profiles differ between pediatric and adult patients. Optimizing outcomes requires personalized surveillance and coordinated multidisciplinary management across all age groups. While MEK inhibitors (MEKi) provide therapeutic benefit, their long-term efficacy and safety, particularly in pediatric patients who may receive these agents for extended periods, warrant further investigation. Additionally, adult patients face unique comorbidities that may complicate therapy. Superficial PNs and potential MEK inhibitor resistance remain underexplored. Growing interest in combination therapies and adjuvant strategies may improve outcomes. Ongoing research is crucial to personalize treatment regimens, to identify effective combinations, and to refine surveillance protocols, ultimately enhancing long-term quality of life for individuals living with NF1-PN.

Key Points

- NF1-PNs cause significant morbidity, requiring personalized, multidisciplinary care.
- MEK inhibitors have transformed treatment for NF1-PNs, but side effects and use vary by age.
- Improving outcomes requires tailored approaches and novel strategies.

Plexiform neurofibromas (PNs) are a hallmark manifestation of neurofibromatosis type 1 (NF1), affecting up to 50% of individuals with this genetic disorder.^1-5^ These tumors arise from the proliferation of Schwann cells and other peripheral nerve sheath components, forming masses that may be diffuse or localized, potentially affecting multiple organs and structures.^1^^,^^2^ NF1-PNs can lead to significant morbidity, including functional impairment, diminished health-related quality of life (HRQoL), chronic pain, and, in some cases, transformation to malignant peripheral nerve sheath tumors (MPNSTs).^2^^,^^6-9^

The management of NF1-associated PNs presents unique challenges across the lifespan, particularly given the differences in age-related presentation and treatment response. This review synthesizes current therapeutic strategies, surgical, pharmacologic, and emerging, with an emphasis on individualized care. A multidisciplinary approach is critical at all stages to optimize outcomes, as clinical decision-making must incorporate age-specific factors such as tumor growth dynamics, comorbidities, and the long-term implications of therapy.

While prior reviews have focused largely on pediatric care, significant gaps remain in lifespan-based management strategies for NF1-PN. This review integrates evidence across age groups to provide a comprehensive framework for pediatric and adult care. It offers expert-informed, age-specific guidance on MEK inhibitor use, addresses toxicity profiles, and incorporates recent regulatory developments and real-world evidence. Underexplored challenges—such as superficial PNs, MEK ­inhibitor resistance, and tumor regrowth after treatment discontinuation—are also discussed. This narrative review aims to provide a clinically meaningful synthesis rather than a comprehensive systematic analysis; as typical of narrative reviews, study inclusion was guided by thematic relevance and expert judgment, which may introduce selection bias.

The review is organized thematically, first outlining the pathophysiology and diagnostic evaluation of NF1-PN, then summarizing the natural history and current management of NF1-PN, and concluding with future directions. Its structure supports the development of personalized, multidisciplinary strategies across the lifespan.

## Literature Search and Review Methodology

Relevant literature was identified through searches of PubMed using combinations of the following terms: “Neurofibromatosis type 1” OR “NF1,” “plexiform neurofibroma” OR “plexiform neurofibromas,” “treatment,” “management,” “therapy,” “therapeutics,” “MEK inhibitor,” “selumetinib,” “binimetinib,” “mirdametinib,” “trametinib,” “cabozantinib,” “surgery,” “resection,” “pediatric,” “child*,” “children,” “adolescent*,” “adult,” and “adults.” Additional publications were identified through review of references in included studies and the authors’ clinical and research expertise. The search included articles published up to June 2025.

## Genetic Etiology and Pathophysiology of NF1-PN

NF1 is an autosomal-dominant disorder affecting ∼1 in 2500 individuals worldwide.^10^^,^^11^ Mutations in the *NF1* gene, which encodes neurofibromin, a tumor suppressor and GTPase-activating protein, cause dysregulation of the RAS/MAPK signaling pathway, leading to uncontrolled proliferation of Schwann cells, fibroblasts, and endothelial cells within the peripheral nerve sheath.^1^ This drives the formation of neurofibromas, including PNs. Preclinical models demonstrate that immune cells (eg, mast cells, macrophages) promote PN pathogenesis by driving inflammation, tumor growth, and tissue remodeling, including vascular ingrowth and collagen deposition.^1^

For additional information on cellular origin and tumor progression, please see Supplementary Text S1.

## Diagnosis, Surveillance, and Monitoring of NF1-PN

Although no single consensus definition of PN exists, diagnosis typically relies on accepted clinical, radiologic, and histopathologic criteria. The definition may vary depending on context and available data, ranging from clinical assessment alone to inclusion of imaging or, less commonly, biopsy.^2^^,^^7^^,^^12-15^ Spontaneous PNs are extremely rare and are almost always associated with NF1,^16^ which is diagnosed according to established consensus criteria (Table 1).^17^ Biopsy is generally unnecessary for PN diagnosis but may be warranted to exclude malignant transformation, particularly when MPNST is suspected on the basis of imaging or clinical progression.^2^

**Table 1.** Revised diagnostic criteria for NF1

Two or more of the following in an individual who does not have a parent with NF1^a^
Café-au-lait macules (≥6) >5 mm in diameter in prepubertal patients >15 mm in diameter in postpubertal patients
Axillary or inguinal freckles
Neurofibromas (≥2 of any type) or 1 PN
Optic pathway glioma
Lisch nodules (≥2) or choroidal abnormalities (≥2)
A distinctive osseous lesion such as Sphenoid dysplasia Anterolateral bowing of the tibia Pseudoarthrosis of a long bone
A heterozygous pathogenic *NF1* variant

Abbreviations: NF1, neurofibromatosis type 1, PN, plexiform neurofibroma.Adapted from Legius et al (2021)^17^ by CC-BY open access license.

a One or more of these criteria are required in an individual who has a parent with NF1.

Comprehensive physical and neurological examinations are essential to detect palpable masses, deformities, or signs of nerve involvement, such as weakness or pain. Regular evaluations should monitor for malignancy indicators, including rapid tumor growth, new or worsening pain, change in tumor texture (eg, hardening), swelling, new neurological symptoms, unexplained weight loss or night sweats.^2^^,^^3^^,^^18-21^ These assessments are particularly important in adolescents and adults, when MPNST risk increases.^2^^,^^3^^,^^8^^,^^18-21^

Non-contrast magnetic resonance imaging (MRI) using a robust fluid-sensitive sequence, such as short tau inversion recovery, is the gold standard for diagnosis and follow-up of NF1-PN, allowing visualization of tumor size, location, and involvement of adjacent tissues.^2^^,^^3^^,^^22^ However, no consensus guidelines exist on imaging frequency, modality, or baseline screening, leading to wide variation in clinical practice.^3^^,^^18^^,^^22^ Most NF specialists will image PNs every 1-2 years or as symptoms occur. This is based on consensus, not prospective data.

In symptomatic pediatric and adult NF1 patients, regional imaging is typically used to assess for tumors based on the location of clinical symptoms or physical exam findings.^3^^,^^22^ Whole-body (WB)-MRI is increasingly utilized for surveillance in certain populations, although its role remains debated.^2^^,^^18^^,^^22-24^ Because MPNST risk rises sharply in adolescence and young adulthood, some experts recommend a baseline WB-MRI in late adolescence or early adulthood, when sedation is less likely to be required.^8^^,^^9^^,^^23-25^ This imaging may help assess internal tumor burden as a means of stratifying MPNST risk. Some retrospective data support WB screening using positron emission tomography (PET)-computed tomography (CT) for asymptomatic patients; however, radiation exposure is a concern given NF1 patients’ elevated cancer risk.^25-29^ Consequently, some providers may opt for PET-MRI, but it is not widely available.

For additional information on PET imaging, please see Supplementary Text S2.

The presence of distinct nodular lesions (DNLs) on imaging may indicate atypical neurofibromatous neoplasm with uncertain biological potential (ANNUBP), which is associated with an increased risk of malignant transformation.^21^^,^^30^^,^^31^ Rapidly growing or newly painful DNLs or ANNUBPs require prompt imaging and biopsy to assess for malignant transformation.^21^ Given the heterogeneity of PNs, biopsies should target the most concerning areas based on imaging, obtaining multiple core samples when malignant transformation is suspected in collaboration with surgery and/or interventional radiology.^2^^,^^32^ When ANNUBP is confidently suspected on imaging with low suspicion for MPNST, primary resection may be considered when safe and possible.^25^

Consensus guidance recommends an integrated histopathologic-molecular diagnostic approach for NF1-associated peripheral nerve sheath tumors—particularly ANNUBP and MPNST—to standardize criteria, guide biopsy targeting, and improve reporting.^32^

A representative imaging illustrating a classic PN, a DNL/ANNUBP, and MPNST is provided in Supplementary Figures S1-S3.

## Natural History and Clinical Impact of NF1-PN

Patients with PNs exhibit a wide variety of clinical presentations, ranging from asymptomatic lesions to those causing significant symptoms.^33^ The clinical course and risk of complications are influenced by factors such as tumor size, location, nerve involvement, and patient age.^33^ While many PNs are congenital, the prevalence of measurable PNs in individuals with NF1 increases with age. In a Danish population study, PNs of any size were identified in 38% of children and adolescents and 64% of adults; for large PNs (≥3 cm), prevalence increased from 12% in pediatric patients to 21% in adults.^34^ These findings emphasize the progressive accumulation of PN burden throughout life.

Growth patterns of PNs vary both within and between individuals. Growth rates of PNs correlate with total WB tumor volume and are inversely related to age; however, the specific drivers of growth and shrinkage remain unclear.^8^^,^^35^ Factors such as tumor location, sex, race, pregnancy, and hormonal fluctuations related to puberty do not appear to significantly impact PN growth, suggesting other, as-yet-unidentified drivers.^9^^,^^35-37^

### Pediatric Course

PNs are typically present at birth but can be newly detected in childhood.^38^ Early in life, NF1-PN may initially be asymptomatic or cause minor cosmetic concerns.^7^^,^^33^ Superficial PNs, which often appear in early childhood without anatomical predilection are typically small, painless, and rarely impair function; however, larger, deeper, or infiltrative lesions may cause pain or functional decline.^7^^,^^33^ Most symptomatic PNs in children are larger in volume and most commonly located in the head and neck region, followed by the extremities and the trunk.^3^^,^^33^^,^^39^ For example, among 65 children with NF1, 73 PNs were detected, 11 in the head and neck region (7 symptomatic), and most thoracic or abdominal tumors were asymptomatic.^33^ Similarly, the National Cancer Institute (NCI) Natural History study found PNs widely distributed, with 44% in the head, neck, or upper chest, and 56% in the trunk or extremities.^40^

Growth is most rapid in early childhood, although it can continue unpredictably into adolescence and early adulthood.^8^^,^^35^^,^^40-42^ Longitudinal data from the NCI (NCT00924196) and the University of Hamburg reported the highest median annual growth rates in children and young adults (12.4% to 14.3%).^8^^,^^9^^,^^35^^,^^41^ In an ongoing natural history study, children aged 3-5 years had particularly high growth rates (35.1% per year), which declined to 13.1% per year by adolescence.^40^

During adolescence, tumor growth generally slows; however, complications related to tumor size, location, and organ involvement often become more prominent.^35^^,^^40^^,^^41^ These changes contribute to an increasing symptom burden, including pain, neurologic deficits, functional impairment, and psychosocial challenges, alongside a rising risk MPNSTs.^18-20^^,^^35^^,^^40^^,^^41^^,^^43^^,^^44^ Most MPNSTs are diagnosed between the late third and mid-fourth decades of life, and over 60% of MPNST-related deaths in NF1 occur before age 40 years.^6^^,^^18^^,^^45^

### Adult Course

PNs in adults often involve the abdominopelvic region, brachial plexus, and lumbosacral plexus, and are challenging to manage because of their proximity to critical anatomical structures.^7^^,^^46^ Adults often have more complex clinical profiles and have a higher risk of malignant transformation.^18^ PNs in adults generally grow less frequently than in children, and, interestingly, spontaneous regression may occur.^35^ In 1 study of 47 adults, 56%-63% of internal neurofibromas and PNs spontaneously regressed by ≥20% over 10 years, while only 17% of tumors grew in that period; the median shrinkage rate was 3.7% per year.^9^ This contrasts with the rarity of regression in children and may reflect senescence of tumorigenic Schwann cells in adults.^47^ Notably, 91% of adults without baseline tumors remained tumor-free at follow-up, suggesting that frequent radiographic surveillance may not be necessary in this group.^9^ However, 61% of adults with baseline tumors experienced growth in at least 1 tumor over time, highlighting the unpredictable nature of tumor growth after adolescence.^9^^,^^35^ This variability may reflect additional molecular events beyond the initial NF1 mutation, such as those described earlier.

Long-term data in adults remain limited as most studies have focused on pediatric disease, resulting in a gap in understanding the disease course later in life.^9^ This is ­compounded by variability in tumor growth patterns, inconsistent imaging, and selection bias toward more symptomatic patients, including spontaneous regression.^8^^,^^9^^,^^35^ Many adults with asymptomatic or stable disease do not undergo routine imaging, leaving gaps in longitudinal tumor burden data.^22^^,^^34^

### Morbidity Across the Lifespan

Across all ages, morbidity varies with tumor size, location, and growth pattern. Common complications include disfigurement, pain, motor dysfunction, visual or hearing impairments, airway obstruction, speech and swallowing difficulties, bowel or bladder dysfunction, and, rarely, hemorrhages.^4^^,^^39^^,^^48^^,^^49^ Tumors involving the abdominopelvic region, brachial plexus, or lumbosacral plexus pose particularly high morbidity risk due to their proximity to critical anatomical structures.^7^^,^^46^ In the NCI Natural History study, 88% of patients experienced at least 1 PN-related morbidity, often emerging early in life and typically requiring intervention.^40^ The most common baseline findings were pain (51%), the need for PN-related surgery (44%), and motor dysfunction (27%).^40^ Over time, more than 52% (30 of 57) of PNs exhibited an increase in associated functional morbidities between baseline and maximum PN volume assessment.^40^ Importantly, the study population had higher tumor and overall disease burden compared to the general NF1 population.^40^ Larger tumors are generally associated with more severe functional impairments, although smaller tumors can still cause significant morbidity depending on location.^40^ Symptomatic PNs and their associated morbidities tend to worsen over time if untreated. Children with symptomatic PNs have a higher mortality rate (3.2%) than those with asymptomatic or no PNs (0.5%).^4^

In addition to these physical and functional complications, pediatric and adolescent patients with NF1-PN may also experience psychosocial morbidities,^50^ which often persist or worsen into adulthood.^51-56^ A detailed discussion of psychosocial morbidity, HRQoL, supportive care, and rehabilitation across the lifespan is provided in *QOL, Supportive Care, and* *Rehabilitation in NF1-PN.*

## Management of NF1-PN

**Figure 1.**
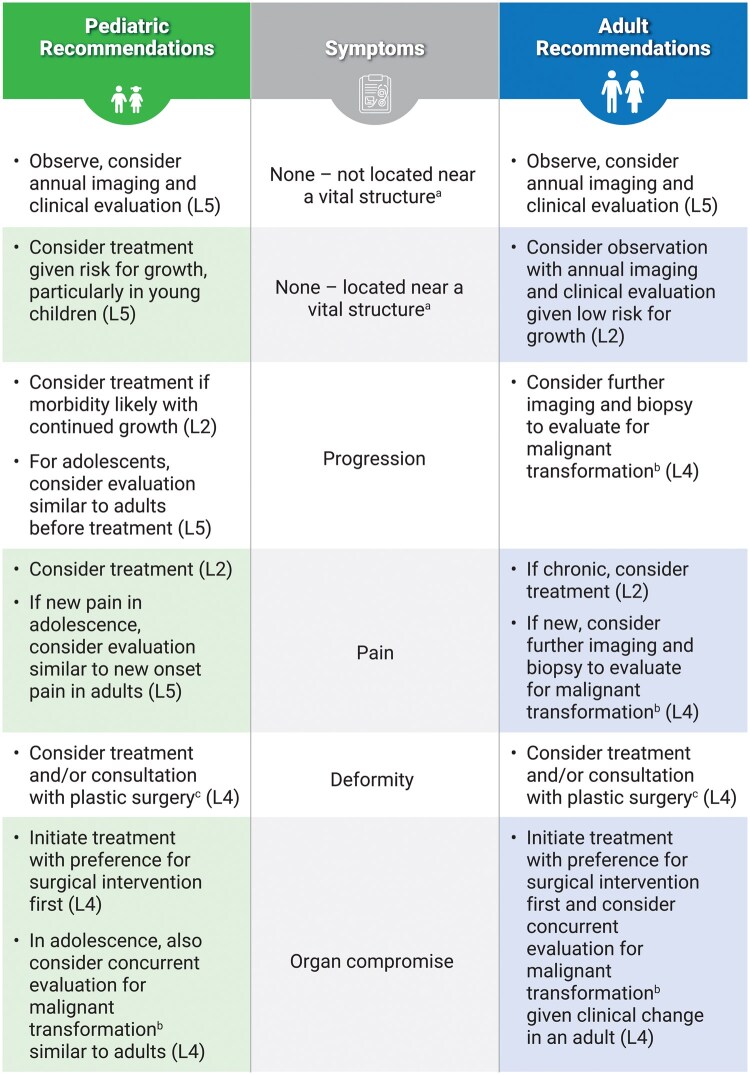
Age-based treatment decision algorithm for patients with NF1-PN. Each recommendation in this algorithm is assigned a level of evidence according to the Oxford Centre for Evidence-Based Medicine hierarchy.^59-61^ L1 = systematic reviews/meta-analyses of RCTs or high-quality RCTs; L2 = lesser-quality RCTs, prospective comparative studies, or cohort studies; L3 = case-control studies; L4 = case series or poor-quality cohort/case-control studies; L5 = expert opinion. Abbreviations: FDG, fluorodeoxyglucose; MRI, magnetic resonance imaging; L, level; NF1, neurofibromatosis type 1; PET, positron emission tomography; PN, plexiform neurofibroma; RCT, randomized controlled trial. ^a^Vital structure examples: eye with vision concern; auditory canal with hearing concerns; trachea with breathing concerns; chest with respiratory or cardiac concerns; abdomen or pelvis with urinary or bowel concerns; spinal cord with neurologic concerns. ^b^Evaluation could include FDG-PET computed tomography or FDG-PET MRI, diffusion-weighted imaging on MRI; abnormal findings on imaging can guide biopsy. ^c^Treatment, including operative management, may be necessary for resection of superficial tumors and soft tissue reconstruction/closure.

Because of their complex growth patterns and infiltrative nature, PNs are often challenging to treat surgically. Effective PN management requires a multidisciplinary team, including geneticists, neurologists, oncologists, radiologists, pain specialists, surgeons, and social workers, to comprehensively address tumor monitoring, symptom management, and psychosocial needs.^2^^,^^3^^,^^57^^,^^58^ Treatment decisions must consider patient age, symptom severity, malignancy risk, and QOL impact, and be tailored to each individual’s physiological and developmental stage.^59-61^ As a result, a treatment algorithm was developed to aid providers with age-based treatment decisions for patients with NF1-PN (Figure 1).

### Outcome Assessment in NF1-PN

The complexity of NF1-PN has driven substantial advancements in clinical trial endpoints. Traditional linear or bidimensional tumor measurements proved inadequate for these large, heterogeneous, and often diffusely infiltrative tumors, limiting sensitivity and clinical relevance.^62^ Volumetric MRI has therefore become the gold standard in phase 2 and pivotal trials, with a ≥ 20% PN volume reduction defining radiographic response.^63^ This threshold has been consistently adopted, including in SPRINT (selumetinib) and ReNeu (mirdametinib), with responses optimally evaluated by independent central review for objectivity.^64^^,^^65^

Volumetric MRI, however, introduces distinct challenges. Achieving consistent measurements across time points is technically complex, particularly for diffuse, infiltrative lesions.^66^ These limitations have fueled debate regarding optimal imaging approaches and outcome criteria, with increasing recognition that imaging alone may not fully capture NF1-PN’s clinical and biological heterogeneity.^62^

To address this gap, secondary endpoints in major trials now incorporate patient-reported outcomes (PROs) and functional measures.^62^ These endpoints assess pain severity and interference, muscle strength, range of motion, and HRQoL.^62^ International collaborations (eg, Response Evaluation in Neurofibromatosis and Schwannomatosis group) have published consensus recommendations advocating for routine use and standardization of PROs, such as the Pediatric Quality of Life Inventory (PedsQL) and disease-specific modules, to ensure comprehensive evaluation beyond imaging.^67-69^

For systemic therapies such as MEKi, radiographic responses are typically gradual, and meaningful symptomatic or functional improvements may occur earlier. As a result, PROs and functional outcomes are essential complements to imaging when assessing treatment benefit. Frequent early imaging is generally discouraged unless clinically indicated to minimize unnecessary interventions and patient burden.

### Surgical Management

Surgery for NF1-PNs is challenging due to tumor location, size, and infiltrative growth.^70^ Complete surgical resection, although rarely feasible, or debulking had been the primary treatments for PNs, particularly for tumors causing functional impairment or cosmetic concerns.^2^^,^^57^ Surgical candidacy must be carefully assessed, as it is not appropriate for every patient. Even with successful surgery, tumor regrowth rates range from 29% to approximately 70%, depending on the extent of resection.^4^^,^^70^ Nevertheless, surgery, when feasible, remains the only potentially curative treatment for NF1-PNs, and it may be associated with acceptable morbidity. Decisions should be individualized, weighing risks and benefits in a multidisciplinary setting. Current guidance suggests close monitoring of DNL, with biopsy or resection considered for lesions that are symptomatic, FDG-avid, or exhibit significant growth (>20% annually in adults).^71^ For preoperative risk stratification and biopsy targeting, FDG-PET remains preferred.^18^^,^^72-74^

MPNSTs require wide-margin excision to reduce the risk of recurrence,^75^ but this may not be achievable due to tumor proximity to critical structures and tumor infiltration.^76^ In contrast, ANNUBP can often be safely and effectively managed with narrow-margin, fascicle-sparing resection that also prevents tumor regrowth.^71^^,^^77^

### Medical Management

For patients in whom surgery is not an appropriate option, medical management is preferred.^57^ Recent advancements highlight the potential of MEKi, a class of targeted therapies showing benefit in treating symptomatic, inoperable NF1-PNs.^65^^,^^78-89^ Several publications have reviewed the use of MEKi in detail.^2^^,^^57^^,^^90^^,^^91^ See Table 2 summarizes current therapeutic options for NF1-PN management.^2^^,^^4^^,^^57^^,^^58^^,^^64^^,^^65^^,^^70^^,^^92-95^

**Table 2.** FDA-approved therapies and surgical options for NF1-PN: efficacy and adverse reactions

Therapy class, drug, and dose	Key clinical activity in PN	Common adverse reactions according to US Package^92^^,^^96^	Primary reference(s)
**MEK inhibitor^a^**			
**Selumetinib** Children ≥1 years of age with BSA of ≥0.40 m^2^: 25 mg/m^2^ BID (max 50 mg BID) Adults: 25 mg/m^2^	**Primary efficacy outcome measure** ORR (per REiNS) and defined as the percentage of patients who experienced ≥20% reduction in tumor volume on MRI confirmed on a subsequent imaging within 3-6 months **ORR** 66% (33/50; 95% CI: 51%, 79%), all confirmed PR **Confirmed ORR by ICR** 44% (95% CI: 30%, 59%)	Peds: Vomiting: 82% Abdominal pain^b^: 75% Diarrhea: 70% Nausea: 66% Stomatitis: 50% Constipation: 34% Rash^c^: 80% Dry skin: 60% Paronychia: 48% Pruritus: 46% Musculoskeletal pain: 58% Fatigue: 56% Pyrexia: 57% Headache: 48% Hair changes^d^: 32%	AstraZeneca Pharmaceuticals LP^96^ Gross et al^64^ Stenger^93^
**Mirdametinib** Children ≥2 years of age and adolescents: 2 mg/m^2^ BID on days 1 to 21 of a 28-day cycle; maximum 4 mg/dose Adults: 2 mg/m^2^ BID on days 1 to 21 of a 28-day cycle; maximum 4 mg/dose	**Primary efficacy outcome measure** Confirmed ORR, defined as the percentage of patients with complete response (disappearance of the target PN) or PR (≥20% reduction in PN volume), assessed by BICR using volumetric MRI per REiNS to be confirmed at a subsequent tumor assessment within 2-6 months during the 24-cycle treatment phase. **Confirmed ORR by BICR** Adults: 24/58 (41%; 95% CI: 29%, 55%) Peds: 29/56 (52%; 95% CI: 38%, 65%)	Rash^e^ Adults: 90% Peds: 73% Diarrhea Adults: 59% Peds: 55% Nausea Adults: 52% Peds: 27% Vomiting Adults: 38% Peds: 39% Abdominal pain^f^ Adults: 24% Peds: 39% Musculoskeletal pain^g^ Adults: 41% Peds: 41%	SpringWorks Therapeutics^92^ Moertel et al^65^
**Surgery**			
N/A	**Indication** Neurologic compromise or impact on vital structures, pain, and disfigurement, with the overall aim of surgery to reduce morbidity and improve QoL^2^^,^^57^^,^^58^ Best outcomes when complete resection is achievable, but this is rare (∼15% of cases)^2^^,^^4^^,^^57^^,^^70^ Recurrence rates: 29% to approximately 70% depending on whether resection is complete or incomplete^4^^,^^70^	Permanent sequelae (mostly neurologic) in 5%-18% of patients^4^^,^^70^ Hemorrhage, infection, nerve injury, pain, scarring, functional/cosmetic impairment, recurrence, limited resectability^2^^,^^57^^,^^58^	Armstrong et al^57^ Bergqvist et al^58^ Fisher et al^2^ Needle et al^70^ Prada et al^4^

Abbreviations: AEs, adverse events; BICR, blinded independent central review; BID, twice daily; ICR, independent central review; MEK, mitogen-activated protein kinase; MRI, magnetic resonance imaging; N/A, not applicable; ORR, objective response rate; peds, pediatrics; PN, plexiform neurofibromas; PPE, plantar erythrodysesthesia; PR, partial response; REiNS, Response Evaluation in Neurofibromatosis and Schwannomatosis.

a Limited to MEK inhibitors with an FDA-approved indication for the treatment of NF1-PN.

b Includes abdominal pain and upper abdominal pain.

c Includes dermatitis acneiform, rash maculo-papular, erythema, rash pustular, rash, urticaria, exfoliative rash, rash pruritic, and rash erythematous.

d Includes alopecia and hair color changes.

e Includes dermatitis acneiform, eczema, maculo-papular rash, pustular rash, dermatitis, erythematous rash, palmar-plantar erythrodysesthesia syndrome, exfoliative rash, skin exfoliation, pruritic rash, papule, papular rash, and macular rash.

f Includes upper abdominal pain, gastrointestinal pain, and abdominal discomfort.

g Includes non-cardiac chest pain, back pain, pain in extremity, neck pain, musculoskeletal chest pain, myalgia, arthralgia, and bone pain.

### Targeted Therapies for the Treatment of NF1-PN

#### Selumetinib

Selumetinib is a MEK inhibitor approved by the FDA for symptomatic, inoperable NF1-PNs in adult and pediatric patients ages 1 year and older,^96^ and it is approved by the European Commission for symptomatic, inoperable PNs in adult and pediatric patients ages 3 years and older.^97^^,^^98^ It is available in capsules and granules administered orally at 25 mg/m^2^ twice daily and continued until disease progression or unacceptable toxicity.

In the Phase 2 SPRINT trial, the investigator-assessed objective response rate was 68% (later amended to 66% after regulatory review), whereas independent central review resulted in a confirmed overall response rate of 44%.^96^

Selumetinib provides benefits beyond tumor shrinkage, including improved function, reduced pain, and better QOL in children.^64^^,^^79^^,^^80^^,^^84^^,^^88^ Long-term follow-up (5 additional years) shows durable tumor shrinkage and maintained pain improvement in children remaining on selumetinib, with a median PFS of 88 cycles (∼7 years).^88^

A Phase 2 trial (NCT02407405) demonstrated confirmed investigator-assessed partial responses (PRs) in 63.6% (21/33) of adults with inoperable, symptomatic, or growing PNs receiving selumetinib, with no disease progression.^89^ Results from the ongoing pivotal Phase 3 KOMET trial (NCT04924608), the first randomized, placebo-controlled study in adults with NF1-PN, showed a significantly higher ORR at cycle 16 with selumetinib versus placebo (19.7% vs 5.4%; *P *= .011) and a median time to response of 3.7 months.^99^ Among patients with baseline chronic pain scores ≥3, selumetinib produced a greater reduction in pain at cycle 12 and higher rates of clinically meaningful improvement from baseline; reductions in chronic pain were also seen in the full analysis set, regardless of baseline pain score. Changes in PlexiQoL scores were not significantly different between arms.

#### Mirdametinib

Mirdametinib is an oral, highly selective and potent, allosteric, CNS-penetrant small molecule MEK inhibitor. It is the first FDA-approved MEK1/2 inhibitor for both adults and children (≥2 years) with symptomatic NF1-PNs not amenable to complete resection, and approved by the European Commission for the treatment of symptomatic, inoperable PN in pediatric and adult patients with NF1.^92^^,^^100^ Mirdametinib is administered twice daily at 2 mg/m^2^ (maximum dose of 4 mg twice daily), with or without food for the first 21 days of each 28-day cycle and is available in both capsule and dispersible tablet forms.

Mirdametinib’s efficacy was confirmed in the pivotal Phase 2b ReNeu study (NCT03962543), which demonstrated a confirmed objective response rate by BICR of 41% in adults and 52% in children with NF1-PN causing significant morbidities.^65^ The confirmed objective response rate increased to 45% in adults and 54% in children when additional confirmed objective responses observed during the optional long-term follow-up treatment phase were included.^65^ Mirdametinib was associated with early and sustained improvements in patient-reported or parent proxy-reported QOL, worst tumor pain severity, and pain interference.^65^^,^^84^^,^^92^^,^^101^

The clinical utility of mirdametinib for NF1-PN is further supported by its inclusion in the National Comprehensive Cancer Network guidelines as a category 2A recommendation for the treatment of symptomatic PNs in adult and pediatric patients (≥2 years) not amenable to complete resection, reinforcing its role as a viable therapeutic option in this setting.^102^

#### Other MEK inhibitors

Several MEKi have shown promise for NF1-PN but are not yet FDA approved. In a Phase 2 trial (NCT03231306), binimetinib achieved PR in 65% (13/20) of adults with inoperable NF1-PN,^83^ and appears reasonably well tolerated and active in children.^87^ Trametinib holds potential in pediatric and adult populations.^82^^,^^86^^,^^103^^,^^104^ In a Phase 1/2 dose-escalation study across a variety of tumor types, 46% (12/26) of children with NF1-PN achieved a PR, with 83% of these responses ongoing at the time of reporting; safety was manageable.^82^ Preliminary Phase 1/2 data in adults with inoperable NF1-PN indicate response rates of ∼50%.^103^

### Tumor- and Therapy-Specific Considerations

#### Tumor response and treatment duration with MEK inhibitors

Both children and adults can benefit from MEKi; however, differences in tumor response, side effects, and long-term tolerance require age-specific management tailored to patients’ physiological and developmental stages. Young children (≤5 years), who are more likely to face rapid PN progression and higher risk of organ compromise, may benefit most from early MEK inhibitor therapy, tend to achieve greater therapeutic benefit and tolerate treatment better than adolescents.^57^^,^^105^ Nevertheless, it remains unclear if initiating treatment in very young or asymptomatic patients is appropriate due to concerns about long-term toxicity and developmental effects. An ongoing prospective clinical trial of selumetinib is assessing this question (NCT06188741).

Tumor shrinkage with MEKi is typically gradual and variable. In the SPRINT trial of selumetinib in children, the median time to initial tumor reduction (≥20% PN volume decrease) was 8 cycles, and the median time to best response was18 cycles.^88^ Similarly, in the ReNeu trial of mirdametinib, the median time to initial tumor reduction (≥20% reduction in PN volume) was 7.8 months for adults and 7.9 months for children. The median time to best volumetric percentage change from baseline was 15.2 months (range, 4.0-40.0) in adults and 13.4 months (range, 4.0-32.7) in children.^65^ Some patients may experience substantial delay before measurable shrinkage is observed. Because medical therapy can take months to show benefit, alternative interventions (eg, surgery) may be necessary for urgent cases involving organ compromise.

Symptomatic or functional improvements, including pain relief and HRQoL gains, frequently precede radiographic changes. In SPRINT and ReNeu, PRO and HRQoL improvements were often observed prior to radiographic changes in children and adults.^65^^,^^88^ Pain relief was sustained for up to 4 years in selumetinib-treated children, and mirdametinib contributed to long-term PRO and HRQOL maintenance and benefits.^65^^,^^88^

While many patients with NF1-PN experience meaningful tumor shrinkage or symptomatic relief, a subset may derive only limited clinical benefit. Resistance to MEKi is uncommon; however, before concluding treatment failure, clinicians should assess factors such as patient adherence or malignant transformation. Research into response variability and resistance mechanisms is needed to inform future strategies, including combination regimens.^106^ Importantly, while tumor shrinkage and symptom relief are typically durable with ongoing treatment, MEKi are not curative, and complete disappearance of PNs is extremely rare.^65^^,^^78-89^^,^^96^

Dose adjustments during MEK inhibitor therapy are primarily guided by clinical response and observed toxicities, not by serum drug concentrations.^91^^,^^107^ Routine drug level monitoring is not standard practice, as therapeutic ranges have not been defined.^108^

The optimal duration of therapy with MEKi remains unclear.^2^ In most clinical trials of MEKi for NF1-PN, treatment durations of at least 2 years are typical. Some patients have continued therapy for over 3 years, and in the case of selumetinib, treatment durations exceeding 5 years have been reported when clinical benefit persists and toxicity remains manageable.^65^^,^^88^ Further studies are needed to guide long-term treatment strategies, including duration and timing of discontinuation.

Tumor regrowth has been reported following selumetinib discontinuation or dose reduction.^105^^,^^109^ The absence of reliable biomarkers to predict tumor regrowth following treatment discontinuation highlights the need for further research. Some patients have demonstrated renewed tumor response after therapy interruption and reinitiation.^90^ This suggests a potential role for intermittent or cyclical therapy, though robust data are lacking. For now, such approaches may be considered on a case-by-case basis. Importantly, for patients who do not achieve meaningful clinical or radiographic benefit from one MEK inhibitor, switching to a different MEK inhibitor is not currently supported by evidence.

#### Anatomical considerations in treatment response

An interesting consideration is whether MEKi exhibit anatomical proclivity in treating NF1-PN, as has been observed with imatinib, a multi-tyrosine kinase inhibitor with a distinct mechanism. In a study of patients with NF1-PN treated with imatinib, 21% of those with head and neck tumors were responsive (defined as >20% reduction in tumor volume), compared with 3% with tumors in the trunk and 0% with tumors in extremities.^110^ To date, there is no indication that tumor location impacts response to MEK inhibitor therapy^88^; however, further research is needed to determine whether MEKi have anatomical preferences or if they are equally effective across all disease sites.

Superficial PNs pose a unique challenge in both clinical management and clinical trial evaluation because they are more challenging to measure using volumetric MRI. These tumors may also be less responsive to MEKi, though the underlying mechanisms are not well understood. There is an urgent need for studies specifically focused on superficial PNs, including the development of novel treatment strategies or combination approaches that might improve their response to MEK inhibitor therapy.

### Safety Profiles of MEK Inhibitors

**Figure 2.**
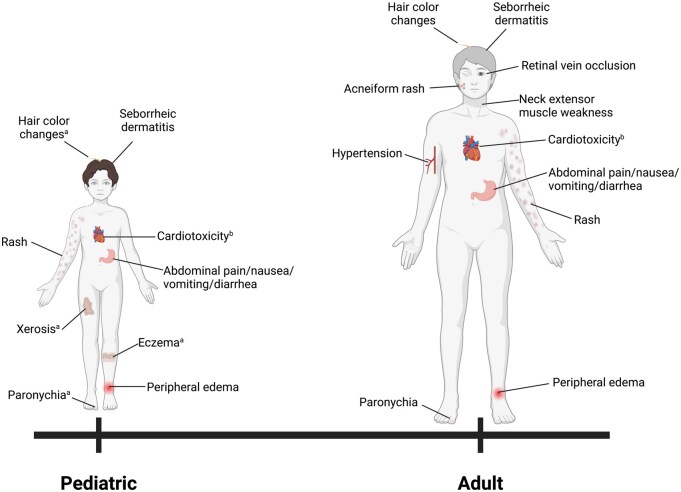
Adverse reactions associated with MEK inhibitors in pediatric and adult patients with NF1-PN. The adverse reactions associated with MEK inhibitors in pediatric patients with NF1-PN overlap with those observed in adult patients. Abbreviations: NF1, neurofibromatosis type 1; PN, plexiform neurofibroma. ^a^Hair color changes, xerosis, eczema, and paronychia have been documented more frequently in children than adults. ^b^Cardiotoxicity, often asymptomatic—such as decreased left ventricular ejection fraction—has been observed in both pediatric and adult patients receiving MEK inhibitors.

MEKi are generally well tolerated but can cause adverse events (AEs) ranging from mild to severe, potentially requiring dose adjustments.^91^ Across agents, safety profiles are generally similar, although the frequency and presentation of individual AEs may vary by drug and patient characteristics, such as age (Figure 2). Creatine kinase elevation is the most frequent laboratory abnormality but is almost always asymptomatic and does not require dose adjustments.^57^^,^^65^^,^^79^^,^^83^^,^^91^ Age-specific supportive care guidelines have been published to guide AE management.^91^^,^^107^

#### Dermatologic toxicities

Dermatological reactions, such as dermatitis, acneiform eruptions, and paronychia, are the most common AEs associated with MEKi and can be quite severe.^79^^,^^87^^,^^88^^,^^91^^,^^111-115^ Distinct patterns have been observed in prepubescent and postpubescent/adult populations. In adults, morbilliform and acneiform eruptions, xerosis, and alopecia predominate.^79^^,^^114^^,^^116^ Postpubescent individuals frequently develop acneiform eruptions and seborrheic dermatitis, whereas younger children (prepubescent and/or under 12 years old) often experience xerosis and eczema-like rashes.^79^^,^^90^^,^^112^^,^^114^^,^^116-118^ These findings suggest that the differences in dermatologic reactions between prepubescent and postpubescent patients may reflect physiological or metabolic changes, which could necessitate different management approaches.^79^^,^^90^^,^^118^

In the ReNeu trial, which included both pediatric and adult populations treated with mirdametinib, a retrospective review of skincare management at a high-enrollment site (*n* = 12; ages 19-68 years) found no need for dose modifications despite dermatologic toxicities.^116^ Consequently, the ReNeu Scientific Steering Committee, with expert dermatologists, developed age-stratified skincare management guidance, addressing differences between prepubescent and postpubescent patients (Supplementary Figure S4).^116^

#### Cardiovascular toxicities

Cardiotoxicity, mainly decreased left ventricular ejection fraction (LVEF), although rare, has been observed in pediatric and adult populations receiving MEKi for NF1-PN. A meta-analysis of oncology trials (not NF1-PN specific) showed a significantly increased risk of LVEF reduction with MEKi versus placebo (odds ratio, 3.35; 95% CI, 1.58-7.07).^119^ A retrospective single-center study of children and young adults treated with selumetinib, trametinib, or mirdametinib found a 6% (6/100) incidence of asymptomatic cancer therapy-related cardiac dysfunction, with a median nadir LVEF of 47.8% and median time to onset of 321 days.^120^

MEK inhibitor-associated decreases in LVEF (even for non-NF1-PN indications) are usually asymptomatic and reversible, often not requiring dose interruption or modification.^65^^,^^79^^,^^88^^,^^121-124^ For example, in the SPRINT trial, 23% (*n* = 17 of 74) of children treated with selumetinib experienced ≥10% decrease in LVEF from baseline; 4% had values below the institutional lower limit of normal, and 1 patient required dose reduction for grade 3 LVEF decline.^96^ All cases were asymptomatic, detected on routine echocardiography, and 71% of cases resolved with treatment discontinuation.^96^ Similarly, the ReNeu trial reported LVEF decreases (10% to <20%) in 25% of pediatric and 16% of adult patients treated with mirdametinib.^65^^,^^92^ Dose interruption was required in 1.8% (*n* = 1) of children and 9% (*n* = 5) of adults with permanent discontinuation needed in 1.7% (*n* = 1) of adults (no children).^65^^,^^92^ In all cases, decreases in LVEF were asymptomatic, identified during routine echocardiography, and resolved in 75% of these patients with continued monitoring.^65^^,^^92^ Symptomatic cardiac toxicity is rare but has been reported in an adult with metastatic melanoma following trametinib use who had no prior cardiac history.^123^

Baseline and ongoing cardiac monitoring with echocardiography is recommended before and during MEK inhibitor therapy in children and adults, in accordance with product labeling, to enable early detection and management of cardiac effects.^92^^,^^96^

Hypertension has also been reported with selumetinib, particularly in adults. A Phase 2 trial showed grade 2 hypertension in 27% (9/33) of adults versus 4% (2/50) of children, with some cases (3/9) representing exacerbation of preexisting hypertension.^88^^,^^89^ Cardiovascular monitoring is essential for patients with preexisting risk factors, such as QTc prolongation, arrhythmias, or hypertension, who may be more vulnerable to these toxicites.^91^

#### Ocular toxicities

Ocular AEs (eg, dry eye, periorbital edema, and retinopathy) are a recognized class effect of MEKi, reported in 5% to 38% of patients.^65^^,^^88^^,^^91^^,^^101^^,^^125^^,^^126^ Retinal events, primarily in adults, often arise within the first treatment month, presenting as blurred vision, photophobia, visual disturbances, and subretinal fluid.^88^^,^^91^^,^^126-130^ Serious AEs (eg, retinal vein occlusion [RVO], retinal detachment) are rare but documented.^65^^,^^101^^,^^126^ RVO has been reported exclusively in adults, including 2 adults with NF1-PN treated with mirdametinib; 1 experienced 2 grade 1 asymptomatic events, and another a grade 3 serious AE resulting in treatment discontinuation.^65^ RVO has not been reported in clinical trials of adults or children with NF1-PN treated with selumetinib.^88^ Most ocular events require no treatment modifications; however, more severe cases may necessitate dose modifications or discontinuation.^88^^,^^91^^,^^92^^,^^96^^,^^126^^,^^127^^,^^130^

Routine ophthalmologic monitoring, in accordance with current product labeling, is recommended before and during therapy in children and adults receiving MEKi.^92^^,^^131^ Because MEK inhibitor-associated retinopathy typically occurs early, especially in adults, enhanced surveillance is advised during the initial treatment phase, with follow-up exams approximately 1 month after initiation and every 3 to 6 months thereafter or as clinically indicated.^91^^,^^127-130^

#### Neuromuscular toxicities

Neck extensor muscle weakness (“dropped head syndrome”) has been reported in adults treated with selumetinib, trametinib, and binimetinib, though none specifically for NF1.^132–134^ In our experience with selumetinib-treated adults with NF1, a few developed this condition, which resolved after a temporary treatment interruption or dose reduction.

#### Long-term and developmental safety

Long-term toxicities remain under investigation.^91^ Dermatologic toxicities are most common with extended use; although typically occurring early, some AEs (eg, skin ulcerations, paronychia) may persist or recur.^88^^,^^91^ Laboratory abnormalities, particularly asymptomatic creatine kinase elevation, also may persist.^88^^,^^91^ Cardiac and ocular toxicities, while rare, may emerge or worsen over time, necessitating ongoing surveillance, particularly in high-risk patients.^91^

In pediatrics, the potential impact of prolonged MEKi on growth and development is a key concern. A multicenter study of prepubertal children treated with selumetinib (median, 1.9 years) demonstrated stable or improved growth velocity and height *z*-scores, with no delays in bone age or pubertal onset.^135^ However, data on longitudinal growth with mirdametinib are lacking, limiting conclusions. Although growth-related AEs were not observed, the absence of formal assessments limits conclusions regarding its effects on pediatric development. Consequently, the long-term impact of extended MEK inhibitor therapy on linear growth and endocrine function remains uncertain, highlighting the importance of ongoing growth monitoring during treatment and long-term follow-up.

#### Management strategies for toxicity

Practical management of MEK inhibitor toxicities includes dose reductions and supportive care as first-line strategies. Clinicians may also use brief drug “holidays” (temporary therapy interruptions) or intermittent dosing schedules with scheduled “on” and “off” periods to improve tolerability, particularly during long-term treatment. For example, selumetinib was efficacious in mouse model of NF1-PN using an intermittent regimen of 5 days on/2 days off; however, further validation is needed (NCT01362803).^79^ Anecdotally, some clinicians have also reported switching between different MEKi in patients who initially responded to treatment, aiming to mitigate nonserious yet intolerable toxicities that could not be managed through supportive care or dose reductions.^136^

### Non-MEK Inhibitor Approaches

Other agents have also been explored for NF1-PN. Cabozantinib—an oral multi-tyrosine kinase inhibitor approved for the treatment of hepatocellular carcinoma, advanced renal cell carcinoma, and thyroid cancer^137^—has also been studied in adults with unresectable NF1-PN. In a Phase 2 trial (NCT02101736), 42% (8/19) of adults (≥16 years) with progressive or symptomatic unresectable tumors achieved PRs, with many reporting reduced pain and improved daily activities.^94^ In pediatrics, cabozantinib primarily stabilized disease, with only 9.5% showing radiographic PRs.^57^

Imatinib has been investigated as a treatment for clinically significant PNs in patients aged 3-65 years. A Phase 2 open-label pilot trial demonstrated disease regression in 26% of evaluable patients, particularly in small (≤20 mL) head and neck tumors. Although these preliminary results are promising, further confirmation in larger, multi-institutional studies is needed.^110^

### HRQoL Supportive Care, and Rehabilitation in NF1-PN

NF1-PN imposes a substantial, lifelong burden on HRQoL, across physical, functional, and psychosocial domains. The presence, size, and visibility of PNs are well-established factors that contribute to increased severity and persistence of psychosocial challenges such as anxiety, depression, stigma, psychosocial stress, and aesthetic prejudice, which often persist or worsen over time.^44^^,^^51–53^^,^^55^^,^^56^^,^^67^^,^^138^^,^^139^ Validated instruments consistently demonstrate significantly lower HRQoL scores in both children and adults with NF1-PN, attributable to pain, physical limitations, emotional distress, and social functioning deficits.^44^^,^^51–53^^,^^55^^,^^67^^,^^138-141^ The impact extends to families, with caregivers reporting reduced HRQoL and productivity loss.^51^

HRQoL challenges evolve across developmental stages, underscoring the need for continuous, age-appropriate supportive care, multidisciplinary management, and proactive transition planning.^18^^,^^37^^,^^54^^,^^142^

#### Pediatric and adolescent considerations

HRQoL impairments often emerge early in life, affecting physical function, emotional regulation, school participation, and peer relationships.^44^^,^^51^^,^^52^^,^^67^^,^^139^^,^^143^ In a real-world analysis, over 60% of children with NF1-PN reported pain and emotional distress, with scores well below normative thresholds.^51^ Supportive care should include individualized pain management, psychosocial interventions, educational accommodations, and early referral to physical, occupational, and speech therapy to address motor, communication, and swallowing difficulties, and foster independence.^44^^,^^67^

Given the lifelong, multisystem nature of NF1, the transition from pediatric to adult services is a critical juncture. Inadequate transition is associated with delayed complication recognition, loss to follow-up, and early morbidity or mortality.^142^ Structured programs, starting in early adolescence and continuing beyond transfer, should integrate direct communication between providers, shared medical summaries, joint visits when feasible, and regular readiness assessments (eg, Transition Readiness Assessment Questionnaire, Neurofibromatosis Type 1-Transition Readiness Assessment Questionnaire).^54^ High-risk individuals with cognitive or psychiatric comorbidities or limited social support require targeted resources and close monitoring.^54^^,^^142^ Resources such as the Children’s Tumor Foundation, Got Transition, and NF1-specific guides for teens and young adults provide valuable support.

#### Adult considerations

In adults, persistent or worsening HRQoL impairments are driven by chronic pain, functional decline, risk of malignant transformation, and psychosocial stressors including employment challenges, sexual self-esteem, and social isolation.^18^^,^^53^^,^^55^^,^^138^^,^^144^ A systematic literature review reported that only 32% of patients with NF1-PN were employed, despite 68% completing high school or some college, reflecting the combined effects of pain, physical limitations, and social stigma on daily functioning and employment.^18^^,^^139^ Limited access to specialized care due to geographic, insurance, or systemic barriers may further compound disease burden.^145^

Rehabilitation should encompass chronic pain management, psychological support, vocational rehabilitation, and postsurgical recovery, with the overarching goals of preserving function, maintaining independence, and supporting participation in community and work life.^54^^,^^142^

## Future Directions

A major challenge in NF1-PN management will be developing individualized treatment strategies that optimize outcomes across the lifespan. Future research should focus on tailoring treatment regimens to tumor biology, genetic markers, and patient age.

### Noninvasive Surveillance and Early Detection

Emerging approaches such as circulating tumor DNA (ctDNA) or cell-free DNA (cfDNA) analysis offer the potential for noninvasive monitoring of malignant transformation from PN to MPNST.^71^^,^^146-148^ Recent studies have shown that cfDNA fragmentomics and ultra-low-pass whole-genome sequencing can distinguish MPNST from PN, while dynamic changes in ctDNA levels may correlate with disease progression and treatment response.^71^^,^^146-148^ Although these methods are not yet standard of care, further studies are needed to define their clinical utility in surveillance and early intervention.

### Expanding Therapeutic Strategies

The role of adjuvant therapy with MEKi in NF1-PN management is actively being explored. Recent studies suggest that a course of MEK1/2 inhibitors following subtotal resection of PNs may effectively prevent rebound growth, compared to subtotal resection alone or adjuvant mTOR inhibitors, with the treatment generally being well tolerated. Additionally, there is growing interest in using MEKi as neoadjuvant therapy to shrink tumors before surgical resection. However, data in this area are still limited,^71^^,^^149-154^ and further studies are needed to determine the efficacy and optimal timing of this approach. While most clinical trials focus on treating tumors already associated with morbidity, preventative therapy—initiating treatment before significant functional impairment—may be a promising avenue to improve long-term outcomes. A secondary prevention trial (NCT06188741) focused on treating high-risk asymptomatic tumors before they cause symptoms is enrolling and may provide valuable insights.

Building on the positive clinical activity of selumetinib and cabozantinib as monotherapies in clinical trials, along with the promising preclinical data suggesting activity at reduced doses and complementary toxicity profiles, a Phase 1/1b/2 trial (NCT06502171) will assess the tolerability and efficacy of their combination in participants aged 16 years and older with progressive and/or symptomatic NF1-PN.

### Targeting Additional Pathways

Beyond MEK inhibition, there is interest in targeting other RAS/MAPK nodes. ERK inhibitors have shown preclinical activity—particularly in combination with CDK4/6 inhibitors—against NF1 tumor models and may help overcome MEK resistance.^155^^,^^156^ These agents remain investigational with no approved role in NF1-PN treatment.

### Novel Agents in Development

HLX-1502, an investigational, oral, small-molecule mitochondrial dysregulator identified through artificial intelligence-driven drug discovery, received FDA Fast-Track designation for NF1-PN. By disrupting mitochondrial energy in tumor cells, HLX-1502 represents a novel therapeutic approach.^157^^,^^158^ It is currently under evaluation in a Phase 2, open-label, single-arm trial (NCT06541847) in patients ≥16 years with progressive and/or symptomatic NF1-PN.

## Conclusion

While treatment options for NF1-PN have advanced, no formal, universally established management guidelines exist. Given the heterogeneity in tumor behavior and patient response, care must be individualized, accounting for differences across pediatric, adolescent, and adult populations. Management typically combines regular surveillance, medical therapies (eg, MEKi), and surgery, with approaches continuing to evolve through ongoing research. Multidisciplinary clinics are essential for accurate diagnosis, informed treatment decisions, and coordinated long-term monitoring for disease progression and treatment-related AEs. Future priorities include developing consensus guidelines, refining treatment protocols, and advancing personalized strategies. Emerging therapies and improved surveillance hold promise for enhancing long-term outcomes and quality of life across the lifespan.

## Supplementary Material

noag023_Supplementary_Data.docx

## Disclaimer

NCCN Guidelines are referenced with permission from the NCCN Clinical Practice Guidelines in Oncology [NCCN Guidelines^®^] for Central Nervous System Cancers V.5.2024. © National Comprehensive Cancer Network, Inc. 2025. All rights reserved. Accessed April 1, 2025. To view the most recent and complete version of the guideline, go online to NCCN.org. NCCN makes no warranties of any kind whatsoever regarding their content, use or application and disclaims any responsibility for their application or use in any way.
