# Supplementary material for: Neurofibromatosis type 1-plexiform neurofibromas: Integrating treatment across pediatric and adult populations
Source: Neuro Oncol. 2026 Feb 5;28(4):847–63. doi: 10.1093/neuonc/noag023 (PMC13128489; doi:10.1093/neuonc/noag023)
Supplement: noag023_Supplementary_Data.docx [file noag023_supplementary_data.docx]

# Supplement

## Supplemental Text 1

**Cellular Origin and Tumor Progression: From Schwann Cell Lineage to Malignant Peripheral Nerve Sheath Tumor (MPNST)**

Plexiform neurofibromas (PNs) are heterogeneous tumors comprising neoplastic Schwann cells and non-neoplastic neurofibromatosis type 1 (NF1)-haploinsufficient cells—mast cells, fibroblasts, perineurial cells, and infiltrating immune cells—that contribute to inflammation, tissue remodeling, and angiogenesis, supporting tumor growth and maintenance.^1-5^ NF1 is a multisystem disorder with variable clinical presentation and progression.^3,6^ Individuals with NF1 are predisposed to benign and malignant tumors, notably cutaneous neurofibromas, PNs, and optic pathway or other low-grade gliomas.^7,8^ Cutaneous and subcutaneous neurofibromas, hallmark tumors arising from small peripheral nerve branches are present in nearly all patients and may be infiltrative, involving multiple nerve branches and tissues.^3^ These tumors do not have a risk of malignant transformation but often cause pain, itching or disfigurement and may be resected if symptomatic.

In contrast, PNs develop in 30%-50% of NF1 patients are usually congenital, slow-growing lesions originating from cranial and large peripheral nerve sheaths.^3,8-10^ These may present as large, multinodular masses, capable of infiltrating and displacing surrounding tissues such as viscera, muscle, bone, joints, and skin.^3,6,9,11,12^ PNs can progressively enlarge, causing functional impairments, including motor weakness, pain, and sensory deficits.^9,11,13,14^

Patients with NF1 have a 10%-16% lifetime risk of developing MPNSTs, aggressive high-grade sarcomas with poor prognosis, most arising from pre-existing PNs.^13,15,16^ Malignant transformation involves additional genetic and epigenetic alterations, particularly loss of tumor suppressor genes in the p19^ARF^-MDM2-TP53 and p16^INK4A^-Rb pathways (e.g., *CDKN2A/B, TP53*), commonly seen in MPNSTs but not in benign PNs.^2,17,18^ Loss of *CDKN2A/B* is a well-recognized secondary genetic alteration, while other recurrent events include mutations or deletions in *TP53* and loss-of-function alterations in polycomb repressive complex 2 (PRC2) components (*SUZ12* and *EED)*.^19-22^ These changes disrupt cell cycle regulation and epigenetic control, driving tumor progression and increasing malignancy risk.^19-22^ Continued research is needed to clarify the clinical implications of these molecular events.

## Supplemental Text 2

Metabolic imaging with positron emission tomography (PET) can complement anatomic imaging to assess suspicious lesions and detect malignant transformation.^23^ ^18^F-fluorodeoxyglucose (FDG) is the most commonly used and clinically available tracer for MPNST evaluation, allowing semiquantitative assessment using standardized uptake values (SUVs)^23-27^; however, false positives may occur due to overlapping uptake between benign PNs and MPNSTs.^9,28^ Whole-body FDG-PET/computed tomography and FDG-PET/magnetic resonance imaging (MRI) may also guide biopsy site selection and detect metastatic disease in patients with MPNST.^29,30^ Alternative PET tracers, including 11C-methionine (amino acid transport), have been investigated in NF1/MPNST; robust evidence supporting routine use of alternative PET tracers (beyond FDG) for MPNST is currently lacking.^23^ Diffusion-weighted MRI also shows promise as a noninvasive technique with high sensitivity and specificity for MPNST detection and may serve as an alternative or adjunct to PET, potentially guiding biopsy, although it requires further validation.^9,31-34^

## Supplemental References

1. Carroll SL. Molecular mechanisms promoting the pathogenesis of Schwann cell neoplasms. *Acta Neuropathol.* 2012;123(3):321-348.

2. Carroll SL, Ratner N. How does the Schwann cell lineage form tumors in NF1? *Glia.* 2008;56(14):1590-1605.

3. Staser K, Yang FC, Clapp DW. Pathogenesis of plexiform neurofibroma: tumor-stromal/hematopoietic interactions in tumor progression. *Annu Rev Pathol.* 2012;7:469-495.

4. Mazuelas H, Carrió M, Serra E. Modeling tumors of the peripheral nervous system associated with neurofibromatosis type 1: reprogramming plexiform neurofibroma cells. *Stem Cell Res.* 2020;49:102068.

5. Yang FC, Staser K, Clapp DW. The plexiform neurofibroma microenvironment. *Cancer Microenviron.* 2012;5(3):307-310.

6. Gutmann DH, Ferner RE, Listernick RH, Korf BR, Wolters PL, Johnson KJ. Neurofibromatosis type 1. *Nat Rev Dis Primers.* 2017;3:17004.

7. Legius E, Messiaen L, Wolkenstein P, et al. Revised diagnostic criteria for neurofibromatosis type 1 and Legius syndrome: an international consensus recommendation. *Genet Med.* 2021;23(8):1506-1513.

8. Prada CE, Rangwala FA, Martin LJ, et al. Pediatric plexiform neurofibromas: impact on morbidity and mortality in neurofibromatosis type 1. *J Pediatr.* 2012;160(3):461-467.

9. Fisher MJ, Blakeley JO, Weiss BD, et al. Management of neurofibromatosis type 1-associated plexiform neurofibromas. *Neuro Oncol.* 2022;24(11):1827-1844.

10. Miller DT, Freedenberg D, Schorry E, Ullrich NJ, Viskochil D, Korf BR. Health supervision for children with neurofibromatosis type 1. *Pediatrics.* 2019;143(5):e20190660.

11. Mautner VF, Hartmann M, Kluwe L, Friedrich RE, Fünsterer C. MRI growth patterns of plexiform neurofibromas in patients with neurofibromatosis type 1. *Neuroradiology.* 2006;48(3):160-165.

12. Patel NB, Stacy GS. Musculoskeletal manifestations of neurofibromatosis type 1. *AJR Am J Roentgenol.* 2012;199(1):W99-W106.

13. Evans DG, Baser ME, McGaughran J, Sharif S, Howard E, Moran A. Malignant peripheral nerve sheath tumours in neurofibromatosis 1. *J Med Genet.* 2002;39(5):311-314.

14. Nguyen R, Dombi E, Widemann BC, et al. Growth dynamics of plexiform neurofibromas: a retrospective cohort study of 201 patients with neurofibromatosis 1. *Orphanet J Rare Dis.* 2012;7:75.

15. Uusitalo E, Rantanen M, Kallionpää RA, et al. Distinctive cancer associations in patients with neurofibromatosis type 1. *J Clin Oncol.* 2016;34(17):1978-1986.

16. Mautner VF, Asuagbor FA, Dombi E, et al. Assessment of benign tumor burden by whole-body MRI in patients with neurofibromatosis 1. *Neuro Oncol.* 2008;10(4):593-598.

17. Mitchell DK, Burgess B, White EE, et al. Spatial gene-expression profiling unveils immuno-oncogenic programs of NF1-associated peripheral nerve sheath tumor progression. *Clin Cancer Res.* 2024;30(5):1038-1053.

18. Cortes-Ciriano I, Steele CD, Piculell K, et al. Genomic patterns of malignant peripheral nerve sheath tumor (MPNST) evolution correlate with clinical outcome and are detectable in cell-free DNA. *Cancer Discov.* 2023;13(3):654-671.

19. Grit JL, McGee LE, Tovar EA, et al. p53 modulates kinase inhibitor resistance and lineage plasticity in NF1-related MPNSTs. *Oncogene.* 2024;43(19):1411-1430.

20. Li W, Hu C, Zhang X, et al. SUZ12 loss amplifies the Ras/ERK pathway by activating adenylate cyclase 1 in NF1-associated neurofibromas. *Front Oncol.* 2021;11:738300.

21. Suresh K, Kliot T, Piunti A, Kliot M. Epigenetic mechanisms drive the progression of neurofibromas to malignant peripheral nerve sheath tumors. *Surg Neurol Int.* 2016;7(suppl 33):S797-S800.

22. Rhodes SD, He Y, Smith A, et al. Cdkn2a (Arf) loss drives NF1-associated atypical neurofibroma and malignant transformation. *Hum Mol Genet.* 2019;28(16):2752-2762.

23. Bredella MA, Torriani M, Hornicek F, et al. Value of PET in the assessment of patients with neurofibromatosis type 1. *AJR Am J Roentgenol.* 2007;189(4):928-935.

24. Brenner W, Friedrich RE, Gawad KA, et al. Prognostic relevance of FDG PET in patients with neurofibromatosis type-1 and malignant peripheral nerve sheath tumours. *Eur J Nucl Med Mol Imaging.* 2006;33(4):428-432.

25. Cardona S, Schwarzbach M, Hinz U, et al. Evaluation of F18-deoxyglucose positron emission tomography (FDG-PET) to assess the nature of neurogenic tumours. *Eur J Surg Oncol.* 2003;29(6):536-541.

26. Ferner RE, Lucas JD, O'Doherty MJ, et al. Evaluation of (18)fluorodeoxyglucose positron emission tomography ((18)FDG PET) in the detection of malignant peripheral nerve sheath tumours arising from within plexiform neurofibromas in neurofibromatosis 1. *J Neurol Neurosurg Psychiatry.* 2000;68(3):353-357.

27. Martin E, Geitenbeek RTJ, Coert JH, et al. A Bayesian approach for diagnostic accuracy of malignant peripheral nerve sheath tumors: a systematic review and meta-analysis. *Neuro Oncol.* 2021;23(4):557-571.

28. Tovmassian D, Abdul Razak M, London K. The role of [^(18)^F]FDG-PET/CT in predicting malignant transformation of plexiform neurofibromas in neurofibromatosis-1. *Int J Surg Oncol.* 2016;2016:6162182.

29. Macpherson RE, Pratap S, Tyrrell H, et al. Retrospective audit of 957 consecutive (18)F-FDG PET-CT scans compared to CT and MRI in 493 patients with different histological subtypes of bone and soft tissue sarcoma. *Clin Sarcoma Res.* 2018;8:9.

30. Brahmi M, Thiesse P, Ranchere D, et al. Diagnostic accuracy of PET/CT-guided percutaneous biopsies for malignant peripheral nerve sheath tumors in neurofibromatosis type 1 patients. *PLoS One.* 2015;10(10):e0138386.

31. Ahlawat S, Ly KI, Fayad LM, et al. Imaging evaluation of plexiform neurofibromas in neurofibromatosis type 1: a survey-based assessment. *Neurology.* 2021;97(7 Suppl 1):S111-S119.

32. Well L, Salamon J, Kaul MG, et al. Differentiation of peripheral nerve sheath tumors in patients with neurofibromatosis type 1 using diffusion-weighted magnetic resonance imaging. *Neuro Oncol.* 2019;21(4):508-516.

33. Ristow I, Kaul MG, Stark M, et al. Discrimination of benign, atypical, and malignant peripheral nerve sheath tumors in neurofibromatosis type 1 using diffusion-weighted MRI. *Neurooncol Adv.* 2024;6(1):vdae021.

34. Wilson MP, Katlariwala P, Low G, et al. Diagnostic accuracy of MRI for the detection of malignant peripheral nerve sheath tumors: a systematic review and meta-analysis. *AJR Am J Roentgenol.* 2021;217(1):31-39.

**Supplemental Figure 1A-E. Classic Deep Intrathoracic PN in NF1 With Associated Dextroscoliosis**

**
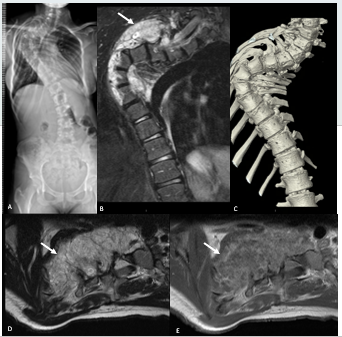
**

A 14-year-old female with NF1 and right paraspinal deep nodular intrathoracic PN (arrow). Note associated dextroscoliosis in standing anteroposterior scoliosis series (A), coronal T2-weighted fat suppressed (FS) MRI (B) and volume-rendered 3-Dimensional computed tomography (C).  Coronal T2-weighted FS (B) and axial T2-weighted (D) and post-contrast T1-weighted (E) MRI through the thoracic spine are optimal for evaluation of PN size, morphology, and anatomic extent.

CT, computed tomography; DNL, distinct nodular lesion; FS, fat suppressed; MRI, magnetic resonance imaging; PET, positron emission tomography; PN, plexiform neurofibroma; SUV_max_, maximum standardized uptake value.

**Supplemental Figure 2. DNL/ANNUBP in NF1**


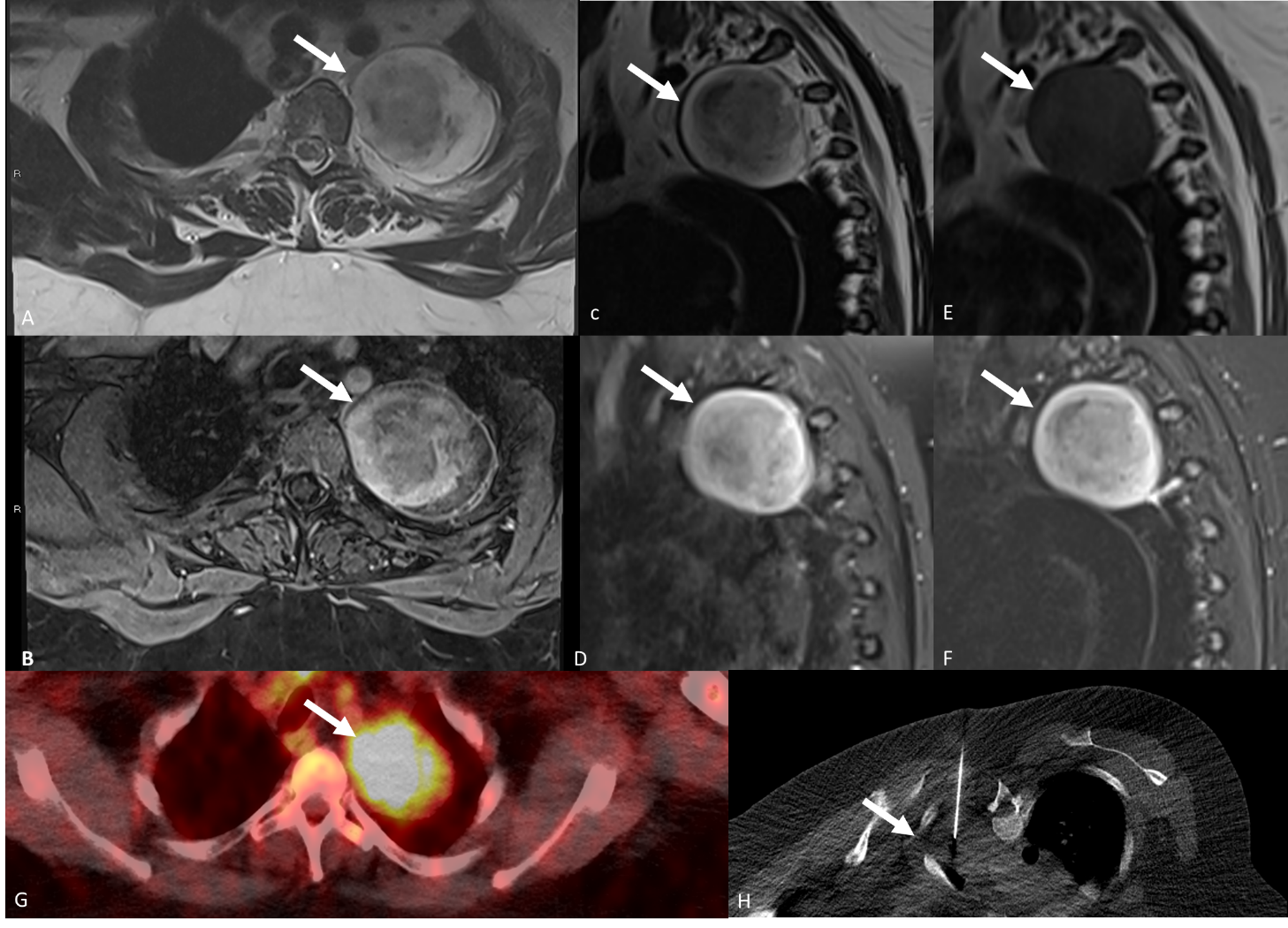


A 41-year-old female with NF1 and left intrathoracic 5 × 4.2 × 4.4 cm distinct nodular lesion (arrow) located lateral to the left T1-T2 neural foramen detected on thoracic spine MRI. Note the lack of a target sign on (A) axial T2- and (B) post-contrast T1-weighted fat suppressed (FS) MRI. (C) Sagittal T2, (D) short tau inversion recovery, (E) T1-weighted non-FS, and (F) post-contrast T1-weighed fat suppressed (FS) MRI depict the anatomic extent of this DNL.  Because of the large size and MRI features, the patient underwent PET imaging (G) which exhibited hypermetabolic activity (SUV_max_ of 19.1). The patient underwent percutaneous CT-guided biopsy (H) and was diagnosed with ANNUBP; however, resection for therapeutic and confirmatory diagnostic purposes is pending.

ANNUBP, atypical neurofibromatous neoplasm of uncertain biologic potential; CT, computed tomography; DNL, distinct nodular lesion; FS, fat suppressed; MRI, magnetic resonance imaging; PET, positron emission tomography; SUV_max_, maximum standardized uptake value.

**Supplemental Figure 3. NF1-Associated MPNST**


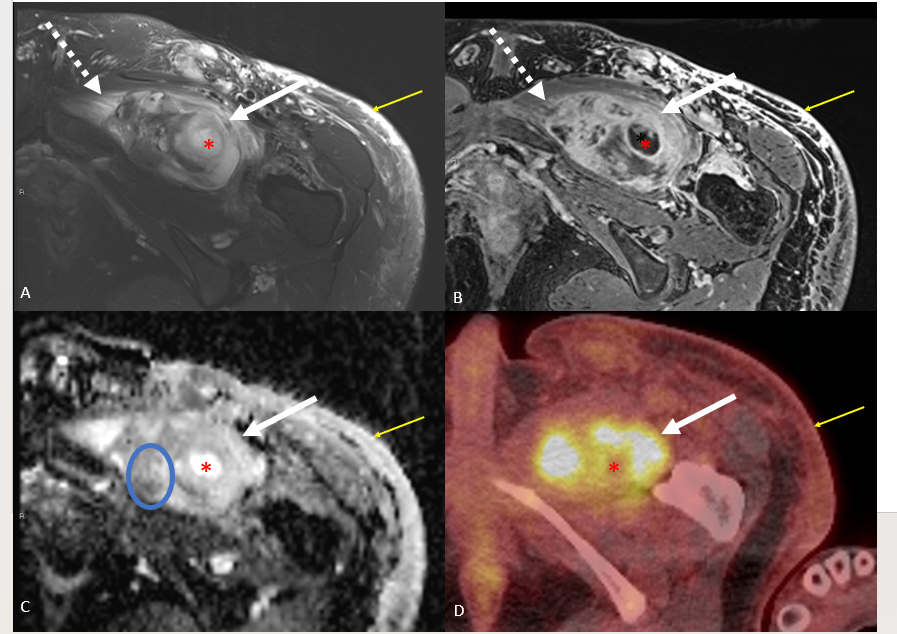


An 18-year-old male with NF1 and deep 8.3 × 4.6 × 6 cm left thigh soft tissue mass (solid arrow) without a target sign.  Note the presence of necrosis (asterisk) and perilesional edema-like signal (dashed arrow on A) on axial T2- weighted fat suppressed (FS) MRI (A). The mass exhibited heterogeneous enhancement on post-contrast T1-FS MRI (B) with perilesional enhancement (dashed arrow on B). (C) On axial ADC mapping, the mass exhibited restricted diffusion denoted by blue oval along the posteromedial margin. (D) On PET imaging, the mass exhibited hypermetabolic activity with SUV_max_ of 5.5. This mass was found to be high-grade malignant peripheral nerve sheath tumor on biopsy. Note the superficial diffuse subtype of plexiform neurofibroma in the skin and subcutaneous tissues of the proximal thigh (yellow arrow) with differing imaging characteristics and morphology than the MPNST.

ADC, apparent diffusion coefficient; FS, fat suppressed; MPNST, malignant peripheral nerve sheath tumor; MRI, magnetic resonance imaging; NF1, neurofibromatosis type 1; PET, positron emission tomography; PN, plexiform neurofibroma; SUV_max_, maximum standardized uptake value.

**Supplemental Figure 4. Skincare Suggestions for All Patients Receiving Treatment With Mirdametinib**


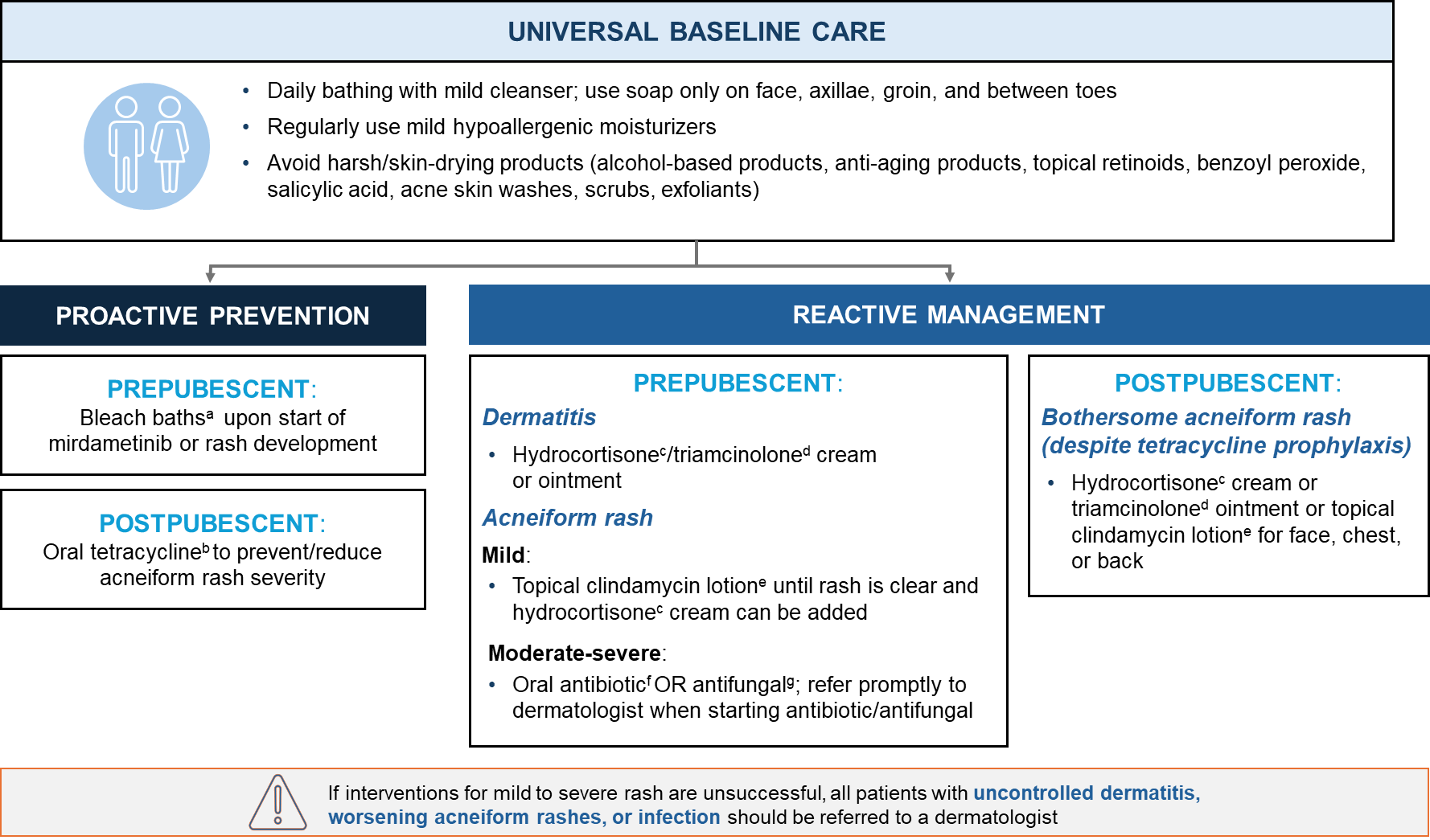


^a^One-quarter to one-half cup of regular strength bleach to a full bathtub (~1 teaspoon per gallon) and soak for 5-10 minutes 3-4× per week.

^b^Tetracycline (eg, doxycycline or minocycline); 50 mg/day for 3 months.

^c^2.5% BID.

^d^0.1% BID.

^e^1.0% BID.

^f^Cephalexin (20 mg/kg/day divided BID; max dose 500 mg) up to 6 weeks or Amoxicillin (25 mg/kg/day divided BID; max dose 875 mg) up to 6 weeks.

^g^Fluconazole (20 mg/kg/day; max dose 100 mg) for 5 days and then 1× per week for 3 months.
